# “Ethereal” Castor Oil

**Published:** 1882-03

**Authors:** 


### "ETHEREAL" CASTOR OIL.

M. Prodam, a pharmacist of Fiume, Austria, has obtained from the castor seeds, by treating them with ether, not only the oil, which would not be purgative by itself unless it contained some of the active principle known as *ricinine*, but a solution which also contains this principle in larger proportion. He is thus able to get the purgative effect of the oil by a smaller dose and without the disagreeable taste of the ordinary oil. His preparation has further the advantage of being miscible, in any proportion, with water, coffee, or milk.—*Chemist and Druggist.*

